# Expanding global distribution of rotavirus serotype G9: detection in Libya, Kenya, and Cuba.

**DOI:** 10.3201/eid0705.017521

**Published:** 2001

**Authors:** N A Cunliffe, W Dove, J E Bunn, M Ben Ramadam, J W Nyangao, R L Riveron, L E Cuevas, C A Hart

**Affiliations:** University of Liverpool, United Kinngdom.

## Abstract

Serotype G9 may be the fifth most common human rotavirus serotype, after serotypes G1 to G4. In three cross-sectional studies of childhood diarrhea, we have detected serotype G9 rotaviruses for the first time in Libya, Kenya, and Cuba. Serotype G9 constituted 27% of all rotaviruses identified, emphasizing the reemergence of serotype G9 and suggesting that future human rotavirus vaccines will need to protect against disease caused by this serotype.


### Expanding Global Distribution of Rotavirus Serotype G9: Detection in Libya, Kenya, and Cuba

**Nigel A. Cunliffe,* Winifred Dove,* James E.G. Bunn,† M. Ben Ramadam,‡ James W.O. Nyangao,§ Raul L. Riveron,¶ Luis E. Cuevas,† and C. Anthony Hart***

*University of Liverpool, Liverpool, United Kingdom; †Liverpool School of Tropical Medicine, Liverpool, United Kingdom; ‡Misurata Teaching Hospital, Misurata, Libya; §Kenya Medical Research Institute, Nairobi, Kenya; and ¶Centro Havana Children's Hospital, Havana, Cuba

Serotype G9 may be the fifth most common human rotavirus serotype, after serotypes G1 to G4. In three cross-sectional studies of childhood diarrhea, we have detected serotype G9 rotaviruses for the first time in Libya, Kenya, and Cuba. Serotype G9 constituted 27% of all rotaviruses identified, emphasizing the reemergence of serotype G9 and suggesting that future human rotavirus vaccines will need to protect against disease caused by this serotype.

Group A rotaviruses are firmly established as the most important etiologic agents of dehydrating gastroenteritis in infants and young children worldwide (1). Severe rotavirus disease is preventable by vaccination. The expected impact of rotavirus vaccines in reducing disease and death from rotavirus infection will be most evident in developing countries, where rotavirus causes up to 500,000 childhood deaths annually (2).

Rotaviruses are nonenveloped viruses whose genome comprises 11 segments of double-stranded RNA (dsRNA), contained in the core of the mature, triple-layered particle (1). Rotavirus serotypes are determined by neutralizing antibody responses to each of the two outer capsid proteins, VP7 (termed G serotype) and VP4 (termed P serotype). Ten G serotypes and 7 P serotypes have been identified in humans (1). Since serotypes G1, G2, G3, and G4 together account for >80% of global human rotavirus strains, some vaccines aim to provide serotype-specific protection against these four serotypes (1). However, in certain geographic settings, other G types (e.g., G5, G8, and G9) may be epidemiologically important (1).

Since their first report in 1987 in the United States, rotaviruses of serotype G9 had been rarely detected in the human population (3). Since 1995, however, serotype G9 has been documented in India, Brazil, Italy, the United States, Bangladesh, Malawi, the United Kingdom, France, and Australia (4). Recent reports from Ireland (5), the Netherlands (6), Japan (7), and Thailand (8) further emphasize the wide geographic distribution of this serotype. We recently characterized rotavirus strains detected during studies of diarrheal disease in children in Libya, Kenya, and Cuba. We identified serotype G9 rotaviruses in each of these countries, providing further evidence that serotype G9 has reemerged as a globally important human serotype.

Address for correspondence: C. Anthony Hart, Department of Medical Microbiology and Genito-Urinary Medicine, University of Liverpool, Duncan Building, Daulby Street, Liverpool L69 3GA, United Kingdom; fax: 44 151 706 5805; e-mail: cahmm@liverpool.ac.uk

### The Study

In all three studies, fecal specimens were collected from children <5 years of age who were hospitalized for treatment of acute gastroenteritis. In Libya, the study was conducted during April and May 2000 and was based at the Misurata Teaching Hospital in Misurata. The samples from Kenya were collected from January to March 2000 from children hospitalized at the Gertrude's Garden Children's Hospital in Nairobi. The third group of samples was obtained during April and May 2000 from children hospitalized at the Centro Havana Children's Hospital, Havana, Cuba.

Fecal specimens were stored at 4°C until they were shipped to the University of Liverpool. Rotavirus infections were diagnosed by negative stain electron microscopy. All rotavirus-positive samples were selected for rotavirus strain characterization.

Rotavirus dsRNA was extracted from fecal samples by a guanidine and silica method (9). The 11 dsRNA segments were separated by polyacrylamide gel electrophoresis and stained with silver to identify the two main RNA profiles, or electropherotypes (long and short). Rotavirus G and P types were determined by using hemi-nested reverse transcription-polymerase chain reaction (RT-PCR). The genotyping methods, which serve as a proxy for serotype determination by virus neutralization, have been described (9,10). A combination of consensus and type-specific primers have been designed to amplify strains of the common rotavirus serotypes, as well as some uncommon serotypes (9-12).

Briefly, for G typing, consensus primers 9con1 and 9con2 were used in a first-round RT-PCR (10 cycles) to generate a 905-bp VP7 gene fragment; 9con1 was then used in a second-round PCR (30 cycles) with type-specific primers 9T-1 (G1), 9T-2 (G2), 9T-3P (G3), 9T-4 (G4), MW8 (G8), and 9T-9B (G9). For P typing, consensus primers con2 and con3 were used in a first-round RT-PCR (10 cycles) to generate a 877-bp fragment of gene 4; con3 was then used in a second-round PCR (30 cycles) with type-specific primers 1T-1 (P[8]), 2T-1 (P[4]), 3T-1 (P[6]), 4T-1 (P[9]), and 5T-1 (P[10]). For strains that failed to give type-specific products, alternative typing primers were used that included a G1-specific primer (nac9) and a P[8]-specific primer (nac10). These primers were designed at the 9T-1 and 1T-1 primer binding regions, respectively, of divergent serotype G1 and P[8] lineages that we have recently identified in Malawi (13). Strains that could not be typed even by using the alternative primers were recorded as nontypeable (NT). RT-PCR products were resolved by electrophoresis on 2% agarose gel and then visualized by ultraviolet illumination after ethidium bromide staining.

Thirty-five (48%) rotaviruses were detected in 73 fecal samples from Libyan children. These comprised strains P[8], G1 (n = 16, 46%); P[8], G9 (n = 12, 34%); P[6], G1 (n = 4, 11%); P[4], G2 (n = 1, 3%); and P[NT], G1 (n = 2, 6%). Seven serotype G1 strains and 23 genotype P[8] strains required alternative primers for efficient typing. Long electropherotype profiles were visualized for serotype G1 and G9 strains, and the single serotype G2 strain had a short profile.

Twenty (41%) rotaviruses from 49 cases of diarrheal disease were characterized from Kenya. The most commonly identified strain was P[8], G1 (n = 12, 60%), followed by P[8], G9 (n = 2, 10%). One strain (5%) of each of the following genotypes made up the rest: P[6], G9; P[4], G8; P[6], G1; P[8], G3; P[NT], G1; and P[NT], G8. Alternative primers were required to type three serotype G1 strains and eight genotype P[8] strains. Overall, serotype G9 was detected in three (15%) of specimens. Most strains, including serotype G1 and G9 strains, had long electropherotype profiles. Both serotype G8 strains had short electropherotypes.

Five (9%) rotaviruses were detected in 55 fecal samples from Cuban children. Three P[8], G4 strains, a single P[8], G9 strain, and a single P[NT], G4 strain were identified. All strains had long electropherotype profiles.

The G9 specificities of representative serotype G9 strains from Libya and Kenya and the single Cuban G9 strain were confirmed by partial nucleotide sequencing of full-length RT-PCR VP7 gene products obtained by using primer pair beg9/end9 (10) (data not shown).

Age data were available for 54 (90%) of the children, including 16 children infected with serotype G9 rotaviruses and 38 infected with other rotavirus strains. The age distribution of serotype G9-infected children (median 15 months; range 6-30 months) was similar to that of children excreting other rotavirus serotypes (median 11.5 months; range 4-57 months).

### Conclusions

These three small cross-sectional studies have documented the presence of serotype G9 in each of the countries surveyed. Serotype G9 was the second most commonly detected serotype in Libya and Kenya, accounting for 34% and 15%, respectively, of G types in those countries. It was also the only G type, apart from serotype G4, to be detected in five samples from Cuba. No previous studies have characterized rotaviruses from either Libya or Cuba. A recent study examined the distribution of rotavirus serotypes in Kenya (14), but to our knowledge this is the first time that serotype G9 has been reported there. Furthermore, this is only the second published report of serotype G9 from Africa, following an earlier report from Malawi (12).

Many serotype G1 and P[8] strains from Libya and Kenya could be typed only by using alternative typing primers, since type-specific products could not be obtained for them with conventional primers. Variation in the sequence of the primer binding region of common serotypes, as well as the occurrence of uncommon serotypes, should be considered when nontypeable rotaviruses are identified in strain characterization studies (13).

Most serotype G9 strains in these studies had long electropherotypes and P[8] VP4 specificity, but a single P[6] VP4 type (also with a long electropherotype) was associated with a serotype G9 strain in Kenya. The reference serotype G9 strains, WI61 (3) and F45 (15), have long electropherotypes and P[8] VP4 specificity. More recently, serotype G9 has been associated with both long and short electropherotypes and a variety of P types including P[6], P[8], and P[11], and molecular characterization of a representative sample of these strains suggests that genomic reassortment played an important role in their evolution (4).

The recent upsurge in published reports of serotype G9 rotaviruses may partly reflect more widespread application of improved methods for their detection. However, increasing evidence indicates that serotype G9 has reemerged in the human population and has recently been imported into certain countries. For example, molecular characterization of serotype G9 strains identified in the United Kingdom demonstrated a high degree of sequence homology among VP7 genes, suggesting that they had been imported relatively recently (16). The observation that children in the United Kingdom infected with serotype G9 strains were older and had more severe disease than children infected with other serotypes supports this hypothesis, since the population would lack neutralizing antibody to this serotype (17). We did not find serotype-specific age differences in this study; however, the numbers may have been too small to allow detection.

More recently, a protracted outbreak of rotavirus diarrhea caused by P[6], G9 strains occurred in a neonatal ward in the Netherlands, which was surprising since rotavirus infection of neonates is typically asymptomatic (6). The unusually high proportion of symptomatic cases may be partly explained by lack of protective, passively acquired maternal neutralizing antibodies to serotype G9 in the affected neonates, since G9 had not previously been identified in the Netherlands (6).

Although our studies are limited by small sample size, we have demonstrated the presence of serotype G9 in each of the three countries, and this serotype represented 16 (27%) of 60 strains overall. These and other recent data suggest that serotype G9 has emerged as the fifth most common global rotavirus serotype. Continued rotavirus surveillance will be necessary to monitor the spread and persistence of this serotype. Future rotavirus vaccines will likely need to provide adequate protection against disease caused by serotype G9 rotaviruses.

### Acknowledgments

We thank Drs. O. Salhuba and P. Mas Bermejo for their assistance.

The Kenyan study was supported by the Japan International Co-operation Agency. Dr. Nigel Cunliffe is the recipient of a Wellcome Trust Research Training Fellowship in Clinical Tropical Medicine (Grant 049485/Z/96).

Dr. Cunliffe is a clinical lecturer in the Department of Medical Microbiology, Liverpool University. He is a Wellcome Trust Tropical Research Fellow working in Blantyre, Malawi, and Liverpool, examining both the diversity of rotaviruses and their effect in HIV-infected children.

### References

1. Hoshino Y, Kapikian AZ. Rotavirus serotypes: classification and importance in epidemiology, immunity, and vaccine development. J Health Popul Nutr 2000;18:5-14.
2. Miller MA, McCann L. Policy analysis of the use of hepatitis B, *Haemophilus influenzae* type B-, *Streptococcus pneumoniae*-conjugate and rotavirus vaccines in national immunization schedules. Health Econ 2000;9:19-35.
3. Clark HF, Hoshino Y, Bell LM, Groff J, Hess G, Bachman P, et al. Rotavirus isolate WI61 representing a presumptive new human serotype. J Clin Microbiol 1987;25:1757-62.
4. Ramachandran M, Kirkwood CD, Unicomb L, Cunliffe NA, Ward RL, Bhan MK, et al. Molecular characterization of serotype G9 rotavirus strains from a global collection. Virology 2000;278:436-44.
5. O'Halloran F, Lynch M, Cryan B, O'Shea H, Fanning S. Molecular characterization of rotavirus in Ireland: detection of novel strains circulating in the population. J Clin Microbiol 2000;38:3370-74.
6. Widdowson MA, van Doornum GJJ, van der Poel WHM, de Boer AS, Mahdi U, Koopmans M. Emerging group-A rotavirus and a nosocomial outbreak of diarrhoea. Lancet 2000;356:1161-2.
7. Oka T, Nakagomi T, Nakagomi O. Apparent re-emergence of serotype G9 in 1995 among rotaviruses recovered from Japanese children hospitalized with acute gastroenteritis. Microbiol Immunol 2000;44:957-61.
8. Maneekarn N, Ushijima H. Epidemiology of rotavirus infection in Thailand. Pediatr Int 2000;42:415-21.
9. Gentsch JR, Glass RI, Woods P, Gouvea V, Gorziglia M, Flores J, et al. Identification of group A rotavirus gene 4 types by polymerase chain reaction. J Clin Microbiol 1992;30:1365-73.
10. Gouvea V, Glass RI, Woods P, Taniguchi K, Clark HF, Forrester B, et al. Polymerase chain reaction amplification and typing of rotavirus nucleic acid from stool specimens. J Clin Microbiol 1990;28:276-82.
11. Das BK, Gentsch JR, Cicirello HG, Woods PA, Gupta A, Ramachandran M, et al. Characterization of rotavirus strains from newborns in New Delhi, India. J Clin Microbiol 1994;32:1820-22.
12. Cunliffe NA, Gondwe JS, Broadhead RL, Molyneux ME, Woods PA, Bresee JS, et al. Rotavirus G and P types in children with acute diarrhea in Blantyre, Malawi, from 1997 to 1998: Predominance of novel P[6]G8 strains. J Med Virol 1999;57:308-12.
13. Cunliffe NA, Gondwe JS, Graham SM, Thindwa BDM, Dove W, Broadhead RL, et al. Rotavirus strain diversity in Blantyre, Malawi, from 1997 to 1999. J Clin Microbiol 2001;39:836-43.
14. Nakata S, Gatheru Z, Ukae S, Adachi N, Kobayashi N, Honma S, et al. Epidemiological study of the G serotype distribution of group A rotaviruses in Kenya from 1991 to 1994. J Med Virol 1999;58:296-303.
15. Green KY, Hoshino Y, Ikegami N. Sequence analysis of the gene encoding the serotype-specific glycoprotein (VP7) of two new human rotavirus serotypes. Virology 1989;168:429-33.
16. Iturriza-Gomara M, Cubitt D, Steele D, Green J, Brown D, Kang G, et al. Characterisation of rotavirus G9 strains isolated in the UK between 1995 and 1998. J Med Virol 2000;61:510-7.
17. Cubitt WD, Steele AD, Iturriza M. Characterisation of rotaviruses from children treated at a London hospital during 1996: Emergence of strains G9P2A[6] and G3P2A[6]. J Med Virol 2000;61:150-54.
